# Round Atelectasis: A Peculiar Pseudotumor Seen on Echocardiogram

**DOI:** 10.7759/cureus.20646

**Published:** 2021-12-23

**Authors:** Nardine Abdelsayed, Larissa Check, Mohamed Faris

**Affiliations:** 1 Internal Medicine, Grand Strand Medical Center, Myrtle Beach, USA

**Keywords:** asbestosis, round atelecatasis, blesovsky’s syndrome, atelectatic pseudotumor, folded lung, pleuroma, pseudotumor, pleural effusion, recurrent pleural effusions, chronic pleural effusions

## Abstract

Round atelectasis (RA) is a rare disorder most commonly occurring in the presence of chronic pleural effusions due to the formation of adhesions and resultant pulmonary collapse. The most common culprit to this disease is asbestosis, but other causes of pleural effusions such as congestive heart failure and pneumonia are reported in the literature. RA can occasionally mimic pulmonary cancers and should be identified to prevent the associated morbidity of tumor workup. We present a case of RA seen on echocardiogram, and then later on computed tomography mimicking a pleural tumor in a 58-year-old female with preexisting heart failure and recurrent pleural effusions. Consultation with radiology and recognition of RA prevented the unnecessary potential morbidity and mortality of further workup.

## Introduction

Round atelectasis (RA) or Blesovsky's syndrome is a form of pulmonary collapse that may appear as a neoplasm on imaging. This atelectasis occurs in the setting of pleural adhesions and thickening with resultant infolding of the pleura and collapse of the underlying lung. Asbestosis is the most documented factor associated with this disease [1]. RA may also occur in the setting of IgG4 pleurisy [2]. Other associations include silicosis [3], sarcoidosis [4], and pleural effusion - occurring more in exudative than transudate effusions [5]. Pulmonary lymphagioleiomyomatosis [6], legionella pneumonia, and other forms of pneumonia such as tuberculosis and histoplasmosis [7-9], congestive heart failure, and end-stage renal disease [10] have also been associated with this phenomenon. We present a case of RA in which invasive workup was avoided due to prior knowledge of common imaging findings of RA in the setting of a pulmonary mass.

## Case presentation

Our patient was a 58-year-old female with a past medical history of type 1 diabetes mellitus, diastolic congestive heart failure with chronic pleural effusions, resistant hypertension, hypothyroidism following thyroid lobectomy (on levothyroxine), tetralogy of Fallot (which was repaired 50 years prior), and coronary artery disease for which she received one stent to the left posterior descending artery six years prior. History was also significant for a low-grade non-invasive papillary urothelial carcinoma which was resected and breast cancer of the left breast post-mastectomy.

She presented to the emergency room with chest pain that began on the morning of admission. The patient mentioned that her pain was similar to the pain she experienced when she had her previous heart attack. She had increasing shortness of breath and bilateral lower extremity swelling over the last month. The patient also had chronic bilateral pleural effusions requiring therapeutic thoracenteses.

In the emergency department, the patient’s blood pressure was found to be 269/143 mmHg with creatinine of 1.9 (normal 0.7-1.5) and borderline elevated troponin of 0.04 (normal 0-0.034) indicating a hypertensive emergency, and her oxygen saturation was 100% on 3 liters of oxygen via nasal cannula. Cardiology was consulted and suspected type II myocardial infarction. No ischemic workup was deemed necessary. Laboratory abnormalities on admission included hyperkalemia at 6.1 (normal 3.5-5.1), hyperglycemia at 228 (normal 74-106) with a hemoglobin A1C of 10.9 (normal <5.6), elevated thyroid-stimulating hormone of 225 (normal 0.45-4.5) with low thyroxine of 0.58 (normal 0.78-2.19), and a brain natriuretic peptide of 8190 (normal <125). Urinalysis showed 300 mg/dl protein.

**Figure 1:**
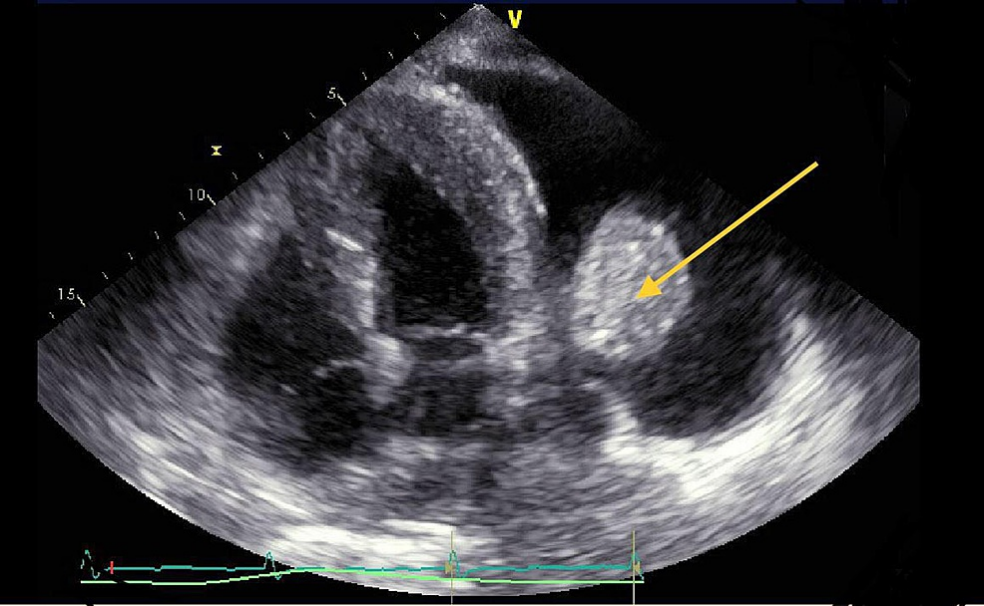
Apical four-chamber view on transthoracic echocardiogram showing mass extending from the pleura (yellow arrow).

**Figure 2:**
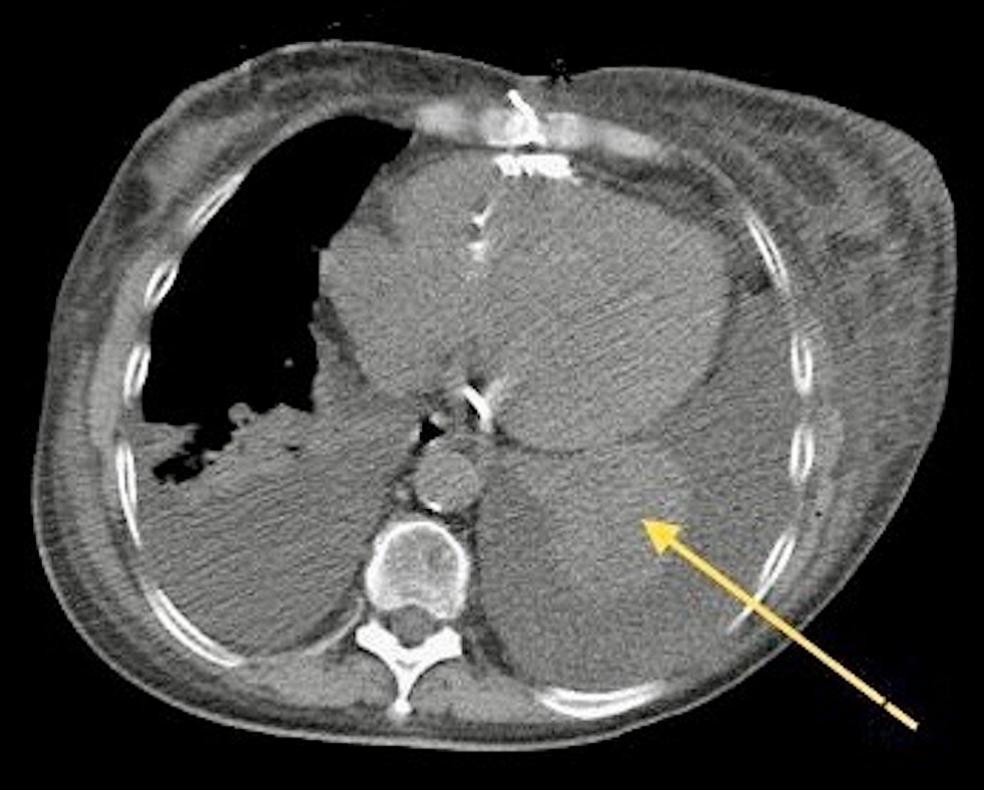
CT thorax showing sub-pleural mass forming an acute angle with the pleura (yellow arrow). CT, computed tomography.

The patient’s chest X-ray showed bilateral pleural effusion more significant on the left side as well as bilateral atelectasis. An echocardiogram was performed to evaluate the current state of her heart failure, and it showed an ejection fraction of 50-55% with no pericardial effusion but noted evidence of a peculiar hyperechoic mass with direct extension to the pulmonary pleura on the left side (Figure 1). A computed tomography (CT) scan of her thorax further visualized this mass, notably the mass was sub-pleural and was forming an acute angle with the pleura (Figure 2). Although the mass was suspicious for possible malignancy, consultation with the radiology department led to the conclusion that this was consistent with the diagnosis of RA, which is an incidental finding associated with her underlying chronic pleural effusions. These findings were also present on prior CT scans, largely unchanged and not contributing to her current symptoms. No further invasive workup was necessary for this incidental pulmonary finding.

The patient was treated for her hypertensive emergency with intravenous (IV) labetalol as well as re-initiation of her home blood pressure medications. These medications successfully brought her systolic blood pressure down to 170 mmHg. The following day, her blood pressure was at goal. Her diabetes was managed with insulin therapy, and she was initiated on an increased dose of levothyroxine. For the patient’s heart failure exacerbation, she was given bumetanide 1 mg IV twice daily, and a therapeutic thoracentesis was performed for her pleural effusion. 

## Discussion

RA is generally asymptomatic and most commonly seen in men (4:1). Occupational exposures such as asbestos are well documented in the literature to be associated with this condition. Due to the stark similarity to lung tumors, recognizing RA may prevent subsequent morbidity of tumor workup such as invasive biopsy or thoracotomy with lobe resection.

Imaging is the hallmark of diagnosis for RA. Magnetic resonance imaging (MRI) and CT thorax with or without contrast are the most commonly used, although ultrasound can also show typical findings. In an MRI, homogenous enhancement is seen, with pulmonary vessels and bronchi converging toward the area of atelectasis, and direct visualization of the infolded visceral pleura is possible in some cases [11]. CT thorax can show similar findings. These ‘masses’ always occur sub-pleural and form an acute angle with the pleura [12]. They are most likely to occur in lower lung lobes [13] and common signs include signs of retraction due to blood vessels and bronchi curving toward the mass (comet-tail appearance) as well as indistinct margins, pleural calcifications, and thickening.

One study following six case reports noted eight major (Table 1 ) and five minor signs (Table 2) of RA [14].

**Table 1:** Major criteria of round atelectasis (present in a majority of cases)

Major criteria
Rounded mass 4-7 cm in diameter lying peripherally in the lung, never completely surrounded by lung tissue
Mass is more dense near its periphery
Mass forms an acute angle with the pleura
Pleural scarring is also present
Comet appearance with vessels and bronchi curving toward the mass
At least 2 sharp margins present
The centrally directed margin is blurred by the entering vessels
An air bronchogram is seen in the central part of the mass

**Table 2:** Minor criteria (only present in some cases of round atelectasis)

Minor criteria
Hyperinflation of the lung adjacent to the mass
Posterior displacement of the right main bronchus (if the lesion is right-sided)
Thickening and displacement of the interlobar fissure (pleural scarring)
Possible bilateral lesions
No change in appearance in 1 year

Unfortunately, some cases have also been reported with coexisting RA and lung cancers such as squamous cell carcinoma and mesothelioma [15] and so clinical judgment must be considered. High-risk clinical features such as extensive smoking history, fevers, hemoptysis, and night sweats should be assessed. In cases where cancer is suspected, a biopsy may be performed to differentiate these diseases. Findings on biopsy in RA include normal lung tissue or abundant pulmonary parenchymal material with thickened alveolar walls containing pulmonary macrophages and connective tissue [16].

## Conclusions

Although rare, RA presents on CT and MRI as a ‘mass’ with direct extension to the pulmonary pleura with distinct features, as mentioned in this study. Physicians should familiarize themselves with this disease entity given its generally benign nature and lack of need for further workup, especially in patients with underlying risk factors such as asbestosis and chronic pleural effusions. High-risk clinical features such as persistent fevers, night sweats, hemoptysis, and extensive smoking history should still prompt a thorough workup and evaluation with biopsy, especially since co-existence with malignancy has been reported. 
